# Characteristics of the Endoderm: Embryonic and Extraembryonic in Mouse

**DOI:** 10.1100/tsw.2006.312

**Published:** 2006-01-19

**Authors:** Mary Familari

**Affiliations:** Department of Zoology, University of Melbourne, Parkville 3010, Australia

**Keywords:** developmental biology, hypoblast, parietal endoderm, visceral endoderm, definitive endoderm, mouse

## Abstract

In mouse, four endodermal lineages are generated during the period from the late blastocyst to the end of gastrulation. The characteristics of each lineage and the proposed genetic cascades involved in their formation are reviewed. In addition, a list of the current markers used to identify these lineages *in vivo* and *in vitro* is presented.

## INTRODUCTION

The ability to derive cell populations from cultured mouse and primate embryonic stem cells (ESCs) has stimulated the research community worldwide. Current research has two main goals. First, these cells offer unlimited opportunities for understanding lineage allocation and differentiation in the early conceptus; these processes have, in most cases, been difficult to study *in vivo*. Second, ESCs offer the opportunity to derive specific cell types for cell-replacement therapies. Much effort is being directed towards the first goal in order to realize the second. Both goals require a thorough understanding of the molecular processes underpinning lineage commitment and differentiation of embryonic cells *in vivo*.

Mouse ESCs[1,2] have full developmental potential, give rise to derivatives of all three primary germ layers *in vitro*, and generate germline chimeras when injected into host blastocysts[3]. Under specific conditions, ESCs differentiate into structures known as embryoid bodies (EBs) that resemble the normal egg cylinder stage[4,5]. The lineages are formed in sequential order resembling the initial developmental stages of the postimplantation conceptus, while prolonged culture leads to spontaneous differentiation in which multiple cell types are generated providing the opportunity to study the underlying molecular processes[6,7].

Although much progress has been made in understanding how the primary germ layers, ectoderm, and mesoderm are formed, the specification and differentiation of the definitive endoderm lineage has only been the focus of more recent attention. The specification and differentiation of the extraembryonic endodermal lineages is also poorly understood. In amniotes, the endoderm presents a unique problem because development of the conceptus involves several “endodermal” lineages, some of which are transient. Embryoid Body (EB) formation *in vitro* allows access to these endodermal lineages[8,9] that are generated in the early conceptus[10,11,12,13,14,15]. It is timely, therefore, to examine the markers that identify the various endodermal lineages in the mouse conceptus *in vivo* and to examine their applicability to identifying these lineages *in vitro*. The focus in this review is mouse development from blastocyst formation to gastrulation, the developmental window during which all four endodermal lineages form.

## LINEAGE ALLOCATION IN EARLY MOUSE DEVELOPMENT

Following fertilization, the mouse conceptus undergoes several rounds of cleavage leading to formation of the morula containing eight cells. Up to this stage, the blastomeres are considered totipotent, although a cell removed at this stage cannot generate a mouse by itself[16,17]. Compaction occurs when the eight blastomeres flatten towards each other increasing their surface contact. The next round of cleavage generates distinct polarized (outer) and nonpolarized (inner) cells that lead to the generation of the first two lineages[17]. These are the outer polarized trophoblast (TB) layer and the inner nonpolarized pluriblast (PB, or Inner Cell Mass), the cluster of cells attached to one region of the TB, depicted schematically in Fig. 1A. The nomenclature of early development in mammals suggested by Johnson and Selwood[18] will be given preference in this review, but alternate terms will be listed as they arise. According to Kunath and colleagues[19], it is not until blastocyst formation that the TB and PB lineages are irreversibly determined, driven by reversible epigenetic changes that differentially occur when polarized and nonpolarized cells are formed during compaction. Just prior to implantation, the PB contains approximately 40 cells, half of which become the hypoblast (HB, primitive endoderm), the third cell lineage to emerge, separating the PB from the blastocoel cavity[20,21]. The HB, a single layer of spindle-like cells, is also the first of four endodermal lineages to appear in the early conceptus (see Fig. 1B).

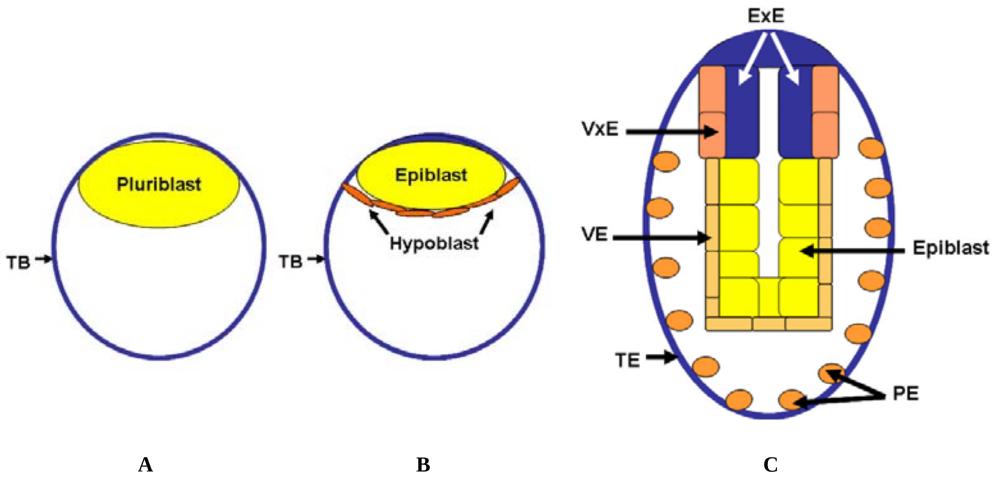

**FIGURE 1.** A schematic representation of early mouse development between the stages of preimplantation and just before gastrulation depicting the location of the extraembryonic endodermal lineages. (A) Preimplantation blastocyst (around 3.5 dpc) showing the first two lineages: TB that will give rise to the extraembryonic ectoderm and PB that will give rise to the epiblast and HB. (B) Just after implantation (around 4.5 dpc), HB, the first endodermal lineage, has formed. (C) Egg-cylinder stage just prior to gastrulation (around 6.25 dpc) showing the location of the visceral endoderm, parietal endoderm, and visceral extraembryonic endoderm - derivatives of the HB. Abbreviations: dpc: days postcoitum, ExE: extraembryonic ectoderm, PE: parietal endoderm, TE: trophectoderm, VE: visceral endoderm, VxE: visceral extraembryonic endoderm.

The TB and HB give rise to different extraembryonic tissues in later development[10,11,12,15,20]. The TB gives rise to several tissues including the extraembryonic ectoderm (ExE), which appears to play an important role in axial patterning[21]. In addition, the derivatives of the TB function as a selective barrier between the fetus and mother leading to the formation of the parietal yolk sac and placenta[12]. The HB has bipotential fate giving rise to both parietal endoderm (PE) and visceral endoderm (VE) that later, in conjunction with contributions from other lineages, give rise to the parietal and visceral yolk sacs, respectively. However, both the precise timing of and processes involved in these events are poorly understood.

Around the time of implantation, PE cells derived from the HB migrate along the mural trophectoderm (TE) generating a discontinuous lining of the blastocoel cavity[13.14] (see Fig. 1C). These isolated PE cells have a nutritive role and secrete a thick extracellular membrane (known as Reichert's membrane) between themselves and the TE, which may function as a filter between fetus and mother[12]. Shortly after implantation, as the polar TE proliferates, it pushes the epiblast into the blastocoelic cavity. Further proliferation leads to the formation of a cylinder of pseudostratified epithelial cells consisting of proximal ExE and distal epiblast (primitive ectoderm). As this cylinder is created, the HB transforms into the VE, lining the entire proximal and distal regions of this structure. The VE varies morphologically in the two regions; the outer layer of the ExE is called the visceral extraembryonic endoderm (VxE) and the outer layer of the epiblast is called the VE (see Fig. 1C). The epiblast gives rise to the three primary germ layers (ectoderm, definitive endoderm, and mesoderm) that contain the progenitors of all the tissues of the fetus and to the extraembryonic mesoderm of the visceral yolk sac, the allantois, and the amnion[10,11,12,13,14,15]. The relationship between the different endodermal lineages is shown in Figure 2.

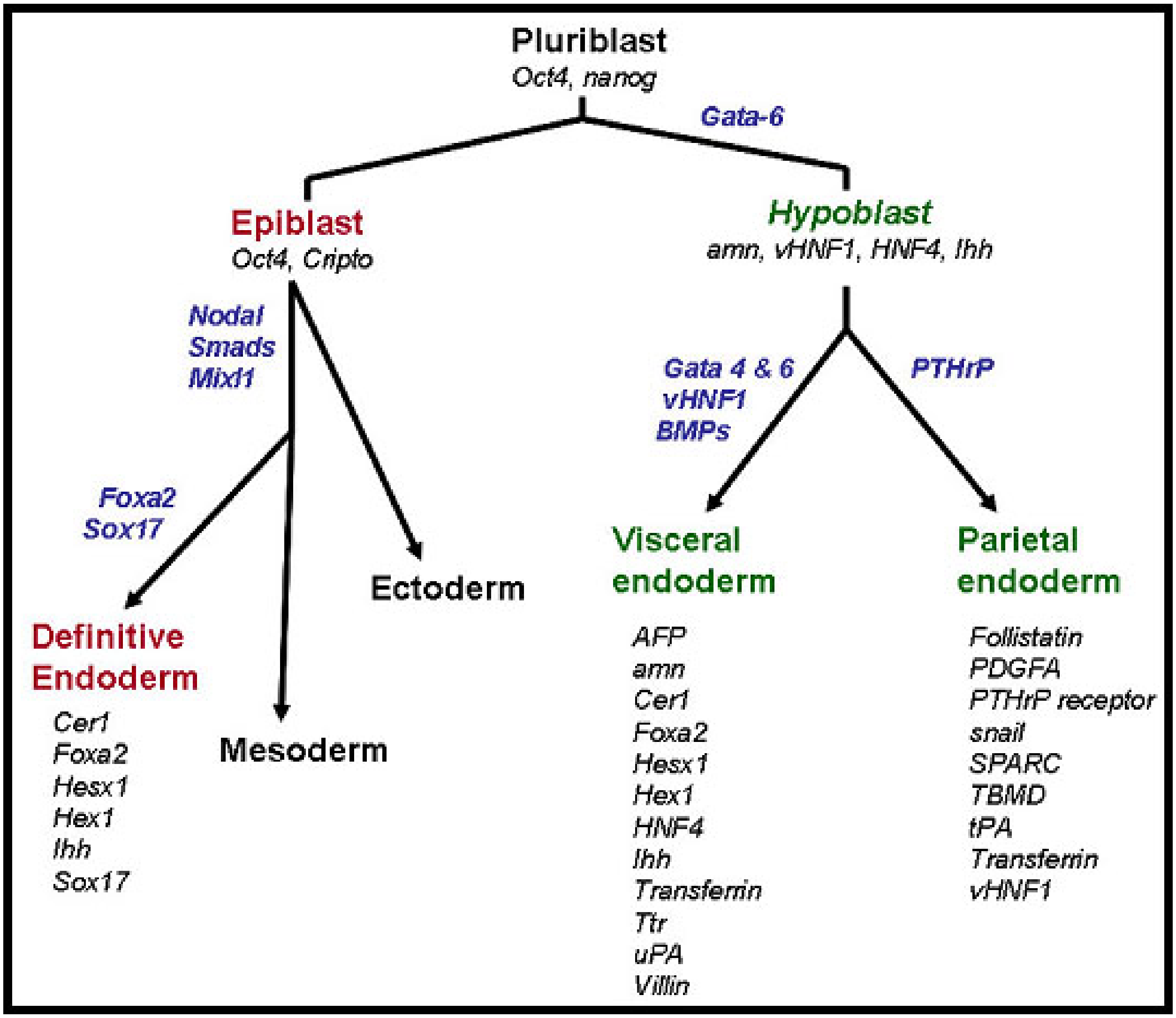

**FIGURE 2.** Diagram showing the lineage relationship between the different endodermal lineages. The factors involved in formation of these lineages are shown in blue. Marker genes for each lineage are shown beneath each tissue

Although PE, VE, and VxE layers are all extraembryonic endodermal tissues derived from the HB, these four layers are morphologically distinct. The HB is not a true epithelium, but forms a reticulum (an irregular layer of cells separating the epiblast from the blastocoel cavity)[13,14]. The cells are considered cuboidal or, more accurately, spindle-like, forming tight junctions laterally and containing extensive endoplasmic reticulum[13,14,15]. In contrast, both the VE and VxE form distinct epithelia, resembling intestinal epithelia morphologically and functionally, containing apical tight junctions and microvilli, as well as vacuoles and organelles distributed asymmetrically. In addition, these cells contain endoplasmic reticulum with enlarged cisternae[13,14,15]. The ability of VE and VxE to adsorb and phagocytose nutrients and proteins has been demonstrated in several studies[14,22,23]. VE also synthesize and secrete transferrin, apolipoproteins, and α–fetoprotein (AFP)[reviewed in 23]. The VE, however, has flat squamous cells containing microvilli while the VxE comprises large columnar cells with more numerous microvilli and very large vacuoles. Dziadek and Adamson[22] demonstrated that these regional differences in morphology were accompanied by differences in AFP expression; the VxE does not express this gene. More importantly, in a further study by Dziadek[24], AFP expression in the VE was inhibited by coculture with ExE, providing some of the first data that extraembryonic tissues affect gene expression.

The VE has an important function in secretion and nutrition during gestation, but it also has an essential role in patterning the early embryo. Mutant phenotypes generating VE dysfunction that implicate this tissue in pattern formation have been reviewed extensively elsewhere[23,25,26,27]. Specifically, reciprocal interactions between the epiblast, VE, and ExE are essential for establishing two signaling centers; one is the anterior visceral endoderm (AVE) and the other is the posterior epiblast[25]. In particular, around 5.5 dpc, a small region of VE at the distal tip of the prestreak conceptus acquires a tall cuboidal epithelial morphology, while the remaining VE remains squamous. These thickened VE cells at the distal tip migrate towards the presumptive anterior position before forming the AVE, which plays an important role in anteroposterior patterning[20,25,26,27]. The organizer in the posterior epiblast directs the formation of the primitive streak, the origin of the primary germ layers including the definitive endoderm[25].

Of interest are several factors expressed in the AVE that are also markers for the definitive endoderm. Prior to gastrulation *Hex1* is expressed in the AVE at the distal tip; as this region of cells migrates anteriorly, they continue to express this factor that plays an important role in the proper expansion of the forebrain region and is later expressed in the definitive endoderm[28]. *Hesx1* is expressed in the AVE at the onset of gastrulation, is expressed in the anterior definitive endoderm by late-streak stage, and by head-fold stage is expressed in both the anterior axial mesendoderm and overlying neural ectoderm where it is also involved in forebrain formation[28]. *Cer1,* expression appears prior to gastrulation in the AVE, extending to the distal tip by the early-streak stage, induces anterior neuroectoderm, and by mid-streak stage, it is restricted to a small patch of endodermal cells overlying the neural plate proximal to the node which includes anterior definitive endoderm[29]. The VE marker, *Foxa2,* that is essential for streak morphogenesis, is expressed throughout the VE around 6 dpc; later expressed in the node, notochord, prechordal plate mesoderm, and anterior definitive endoderm[30].

The formation of the PE generated from the HB just after implantation is viewed as one of the earliest examples of an epithelial-mesenchyme transition in murine development[31]. PE cells are considered to be terminally differentiated. In contrast, VE is able to transdifferentiate into PE[14] and has been demonstrated to contribute cells to the PE up to 8 dpc[32]. In this latter study, an inhibitory effect on transdifferentiation by extraembryonic mesoderm was also reported[32]. The PE is composed of single, nonpolarized migratory cells containing bulky endoplasmic reticulum with enlarged cisternae that synthesize an enormous amount of protein including type IV collagen, laminins, entactin, heparan sulfate proteoglycans, and secreted protein acidic-rich in cysteine (SPARC). These proteins become incorporated into Reichert's membrane, the thick layer of extracellular matrix that forms between PE cells and the TE[14,15,31,33]. The PE is characterized by high levels of tissue plasminogen activator (tPA)[34,35] and the anticoagulant thrombomodulin[36]. A role for this latter factor has not been established.

## FORMATION OF THE ENDODERMAL LINEAGES

### Hypoblast

Several recent publications provide excellent reviews of important transcription factors with demonstrated roles in specification of the first three lineages in the early blastocyst[19,20,37]. Based on null mutation studies and several key observations — in particular that conditional inhibition of *Oct-4* in ESCs leads to TB differentiation[38], *nanog* -/- ESCs differentiate into extraembryonic endoderm[39], and normal ESCs can be derived from *Cdx-2*-/- embryos that lack TB[40] — it has been concluded that lineage allocation in the late blastocyst involves not only the activation of some factors, but also that these factors regulate each other[19,37,40]. TB formation depends on down-regulation of *Oct-4* and gain of *Cdx-2* expression; *nanog* and *Oct-4* are both required for self-renewal and pluripotency of the epiblast, but equally importantly, *nanog* suppresses a HB fate in these cells. *Nanog* and *Cdx-2* have region-specific expression in the late morula, being confined to inner or outer cells, respectively, but what remains unknown is how the expression of any of these three factors becomes restricted[37,40].

Rossant and colleagues[40] have proposed an elegant model for the formation of the HB, which integrates the inside/outside hypothesis, cell division order, and gene expression data without relying on segregation of maternal determinants or segregation of determinants by asymmetric cell divisions. The proposed genetic cascade is as follows. At the 16 cell stage, *Gata-6* is expressed in the outside (polar) cells, presumably as a result of inducing signals from the inner nonpolarized cells. The next round of cleavage forms the blastocyst. The asymmetric cleavage of the outside cells generates both polarized and nonpolarized cells that all express *Gata-6*. The nonpolarized *Gata-6* positive cells become scattered among the *Gata-6* negative nonpolarized PB cells. A further cell division leads to complete down-regulation of *Gata-6* expressing cells in the polarized TB cells. By 4.0 dpc, *Gata-6* positive cells within the PB are sorted to the presumptive HB layer. Finally by the late blastocyst stage, the outer layer of the PB differentiates into HB, which is the only lineage at this stage expressing *Gata-6*. Based on the observation that *Grb2* null mutants lack both HB and expression of *Gata-6*, these authors have suggested the genetic cascade is initiated by FGF, and that *Gata-6* is activated via components of tyrosine kinase signaling including the signal adaptor protein Grb2[40].

It is intriguing that the entire genetic cascade and morphological changes can be accounted for without recourse to segregated determinants. It is proposed that position alone within the morula determines fate, giving rise to polarized and nonpolarized cells. This is strengthened by the propensity to change fate should a blastomere be taken from one of either starting location (inner or outer) and placed in the opposite position during experimental manipulation. It is possible that the generation of polarity in the outer blastomere in itself sets up asymmetric localization of activators and/or repressors of the transcription factors at the head of the genetic cascade. This seems entirely feasible given that there is a large body of evidence attesting to the notion that changes in cell shape can lead to changes in cell function[41], supporting the concept that when a blastomere changes shape from a nonpolarized to a polarized cell, a change in structure can activate a program leading to cell fate. This begs the question, however: What activates the genetic program for cell fate in the nonpolarized inner cell? *Nanog* appears to be activated in the inner nonpolarized cells around the same time that *Cdx-2* expression appears in the outer polarized cells. Therefore, a change in blastomere structure cannot account for the activation of *nanog* in a cell that does not undergo polarization. What is also not clear is whether the change to a polarized cell is the cause or the effect and how this happens. In addition, how are the factor(s) located in the inner nonpolarized cells of the 16-32 cell conceptus activated in order to induce *Gata-6* expression in the outer nonpolarized cells? Perhaps a detailed analysis of the function and duration of the 550 unknown proteins identified in the mouse oocyte may shed light on how *Cdx-2* and *nanog* are activated[42]. At least one maternal determinant, *mater,* is known to be expressed from the one-cell to the late-blastocyst stage in mouse[43].

## Parietal and Visceral Endoderm

Little is known about the precise timing and factors involved in the formation of the PE and VE. In part, this is due to the relatively small size and inaccessibility of these tissues in the mouse conceptus. *In vitro* models to study the differentiation of these tissues, developed several decades ago, are still in use[4,31,44]. In particular, F9 teratocarcinoma cell lines (F9 cells) can be induced to differentiate into VE or PE cells bearing remarkable resemblance to these tissues morphologically, as well as by the expression of tissue-specific genes. Cultured F9 monolayers treated with retinoic acid (RA) and dibutryl cAMP differentiate into PE[45]. Alternatively, when cultured in suspension in medium treated with RA, the cells aggregate forming EBs containing an outer layer of VE. On further treatment with dibutryl cAMP or plating on fibronectin, these VE cells can differentiate into PE[44]. In addition, ESCs treated in a similar manner to F9 monolayers with RA and dibutyrl cAMP can be induced to differentiate into PE.

Expression of tPA, a specific marker for PE, was correlated with PE outgrowth suggesting that degradation of the extracellular matrix (ECM) by this factor facilitated the migratory nature of this cell type[46]. The observation that plating of F9 cells on laminin or fibronectin promoted migration of PE-like cells[47] led to an investigation into whether the ECM triggers PE differentiation or just migration. This important question was addressed by deleting β1-integrin in F9 cells. Adhesion and migration were inhibited in F9 cells, but β1-integrin deficiency did not prevent dibutyrl cAMP–induced differentiation. The authors concluded that matrix adhesion was not essential for PE differentiation[47]. One of the first genes activated on PE differentiation is parathyroid-related Type I peptide (PTHrP) transmembrane receptor, the subject of a recent review[31]. On activation of the PTHrP receptor, the differentiation of PE appeared to be regulated by the interaction of downstream signals with cell-cell/cell-matrix interactions. This is not surprising given that PE formation involves a transition from an epithelial to a migratory mesenchyme-type cell. mRNA and protein for the ligand PTHrP have been demonstrated in both HB and TB giant cells. When added to RA-treated F9 cells, PTHrP was an efficient replacement for dibutryl cAMP in inducing full PE differentiation. Interestingly, PE differentiation was accompanied by an increase in thrombomodulin[31]. However, neither PTHrP nor Type I PTHrP receptor null mutants lack PE, suggesting these factors are not essential for the formation of this tissue *in vivo*[48]. This conclusion has been confirmed by two further observations. First, Type I PTHrP (-/-) ESCs in the presence of RA can form PE. Second, TB-conditioned medium is more effective than pure PTHrP protein alone in inducing the formation of this tissue[31]. The factor(s)/genetic cascade essential for specification of PE remain largely unknown. Obviously, other as-yet-unidentified factors are also required and a concerted effort in this research area will lead to a better understanding of PE specification and differentiation.

A number of targeted mutagenesis studies have identified factors that play an important role in VE formation. *vHNF1*-deficient mouse embryos have abnormal or absent VE, suggesting that this factor is essential for VE specification[49]. In contrast, homozygous mutants involving any of the following genes — *Gata 6*, *HNF4*, *Foxa2,* and *Ihh* — lead to defects in VE differentiation[23]. Although it remains unclear what the temporal and spatial relationships are between these factors, given their critical role in HB formation, *Gata* factors are likely to play an important role at the head of the genetic cascade that results in VE formation.

Both F9 cells and ESCs have been used to develop models of VE differentiation[50,51,52,53]. Although ectopic expression of *Gata 4* and *6* are both capable of driving pluripotent ESCs towards either PE or VE differentiation depending on culture conditions[52], neither *HNF4* nor *Foxa2* alone are capable of doing so. Overexpression of either *vHNF1*[53] or *Ihh*[50] in cultured cells, however, did lead to the induction of some markers of VE differentiation including AFP, but morphologically they did not resemble VE differentiated cells.

In a recent study, several strategies were undertaken to tackle the genetic cascade involving the *HNF* family members in VE formation[53]. First, expression of VE-specific markers was identified in chronological order during induction of the VE. Second, upstream and downstream targets of *vHNF1* were identified in stably transfected cell lines. A genetic cascade leading to VE differentiation was proposed based on the factors identified in this extensive study[53]. At the head of the cascade are the

*Gata* factors and *vHNF1* isoforms involved in a synergistic interaction. *Gata* factors activate *HNF4*, while *vHNF1* activates *HNF1*, *HNF6*, and *Ihh*.

This model[53], allowing for a reciprocal regulative relationship involving *Gata 4* and *6*, sits comfortably with the model of HB formation involving *Gata 6* outlined by Rossant and colleagues[40] and the following observations. *Gata 4-/-* mutants display increased *Gata 6* expression, *Gata 4* expression is absent in *Gata 6-/-* animals, thus *Gata 6* is considered to be an upstream regulator of *Gata 4*[52]. Ectopic expression of either *Gata* factor in ESCs, however, leads to the activation of the other *Gata* gene, suggesting a reciprocal relationship that also allows for redundancy.

In addition, using *in vitro* models, a role for Bone Morphogenetic Proteins *(BMPs)* acting via *HNF4*[54] and *Ihh*[51] in VE differentiation has also been demonstrated. An important role of *BMPs* in VE differentiation is strengthened by the observations that null mutants for these genes (*BMP-2*, *BMP-4*, and *BMP receptor I*) have abnormal VE[28]. Where *BMP-2* and *4* should be placed in the genetic cascade proposed by Haumaitre and colleagues[53] is not entirely clear, but since *BMP-4* is expressed only in the epiblast before HB formation and also induces *HNF4* expression, it may need to be placed upstream of the *Gata* factors or in a synergistic/reciprocal interaction. An analysis of the expression of *Gata 4* and *6* in *BMP* mutants (*BMP-2-/-, BMP-4-/-,* and *BMP receptor I-/-*) and the expression of the *BMP* factors in *Gata* mutants in the late blastocyst at the time of HB formation may help to resolve this issue. The relationship of the *BMPs* to *vHNF1* also remains to be determined.

A complete genetic cascade cannot be derived as not all the factors involved in VE formation are known, although the recent derivation of an extraendodermal stem cell line (XEN cell line) that displays characteristics of both PE and VE has brought this goal a large step closer[55]. The XEN cell lines characterized by microarray gene expression analysis have confirmed many known factors involved in formation of the VE and PE lineages as well as revealing new, previously unknown genes. Curiously, the XEN cell lines display gene expression profiles characteristic for both PE and VE, are highly motile, and the cells undergo reversible distinct morphologies, either round or epithelioid, suggesting a more parietal cell phenotype. Although the authors suggest that different culture conditions may lead to differentiated cells of the VE[55], this has yet to be reported. XEN stem cell lines appear to have the same developmental potential as F9 cells described above, presumably without the obvious disadvantage of genetic lesions.

## Definitive Endoderm

Gastrulation begins around 6.5 dpc as cells in the posterior epiblast delaminate to become the loose migratory mesenchyme of the primitive streak. Fate-mapping studies have demonstrated that endoderm precursors are located in the posterior half of the epiblast at pre- and early-streak stages; they are restricted to the anterior primitive streak and node by mid- to late-streak stage, but as the streak reaches its maximal extension, no more endodermal progenitors lie in the epiblast, primitive streak, or node[11,56,57,58,59].

Both the endoderm and mesoderm progenitors ingress through the anterior region of the primitive streak[11,56,57,58,59,60,61]. Fate mapping shows that the endoderm expands anteriorly and laterally from the area immediately subadjacent to the anterior primitive streak about 10–12 h after the onset of gastrulation, coincident with the exit of cranial and lateral mesoderm progenitors[56,59]. From a correlation of fate maps, it is presumed that endoderm and mesoderm comigrate during gastrulation, although how these lineages are sorted is not known[20,62]. Several studies have demonstrated that definitive endoderm progenitors recruited early in gastrulation are allocated to the anterior region of the embryo subsequently populating the foregut, whereas those cells recruited later populate the posterior region of the gut[11,56,57,59]. It has recently been demonstrated that endoderm progenitors of cells allocated to specific regions of the embryonic gut are already spatially organized in anticipation of the prospective mediolateral and anterior-posterior destinations by late gastrulation[59]. The definitive endoderm progenitors completely displace the VE, which migrates to form the endodermal layer of the visceral yolk sac, although some descendents of the VE are still present in the foregut endoderm of the late-streak embryo[11,57].

By the end of gastrulation, all three primary germ layers (ectoderm, mesoderm, and definitive endoderm) have formed[15,21]. Although the transition from epiblast to ectoderm initially does not involve a change in morphology, the definitive endoderm changes from a columnar epithelium with dense microvilli on the apical surface to a squamous epithelium with sparse microvilli[63]. Several studies have shown that lineage potency is not irreversibly restricted following germ-layer formation. Analysis of heterotopically transplanted cells to other regions in the gastrula have revealed a remarkable plasticity in the lineage potency of epiblast, mesoderm, and primitive streak cells, although the lineage potency of endodermal progenitors has not been tested[56,61].

The molecular and signaling pathways that specify and pattern the endoderm are the subject of several excellent reviews[62,64,65,66,67,68]. How these genetic cascades translate into definitive endoderm is not completely understood, but a great deal of progress has been made in understanding this process in mouse based on studies of animals carrying homozygous deletions in various genes followed by analysis of mutant ESC contribution to wildtype/mutant hybrid embryos. In mouse, the gene-regulatory pathway involved in endoderm formation is activated by a gradient in *Nodal* activity that specifies mesendodermal precursors[66]. Given that the hypomorphic allele of *Nodal* is associated with a complete lack of definitive endoderm, *Nodal* is an essential factor in this pathway[69]. Null mutations in the downstream factors *Foxa2*[30] or *Sox17*[70] lead to loss of foregut or hindgut endoderm, respectively. In mouse, *Sox17* is first expressed in the entire VxE nearest to ectoplacental cone around 6.5 dpc and is also expressed in definitive endoderm until 8.5 dpc. Curiously, in *Sox17-/-* animals, the putative endoderm region is populated by cells having the morphological and functional characteristics of the VE. In chimeras, *Sox17* mutant ES cells can contribute to some extent to foregut but not mid- or hindgut. In contrast, *Foxa2* null ES cells can form hindgut, but not fore- or midgut, suggesting endoderm is specified, but gut morphogenesis is defective in *Foxa2* or *Sox17* null mutants. As mentioned earlier, many genes that are expressed in the AVE are also expressed in the definitive endoderm. Of interest are those genes that have been used to identify definitive endoderm in studies attempting to derive this lineage from embryoid bodies *in vitro*, including *Hex*, *Hesx1,* and *Cer1*.

## MARKERS FOR THE ENDODERMAL LINEAGES

Table 1 lists the markers in most common use to detect the endodermal lineages in both *in vivo* and *in vitro* studies. This is the first instance that a list of expression markers for the different endodermal lineages has been presented with their reported expression pattern in the early conceptus. Three conclusions can be drawn from this table. First, the HB is poorly characterized. Second, the full expression profile of many markers is unknown. Third, for those that are known, and even for those that are partially known, expression patterns overlap in different tissues even at the same stage of development. Overlapping expression patterns may make it difficult to identify particular lineages during EB formation, but also may have implications when attempting to ascertain what lineages are formed in mutant embryos following a particular gene deletion. It is presumed in EBs derived from wild-type ESC that the lineages are formed in sequential order resembling those that form during the egg-cylinder stage. However, it becomes crucially important to distinguish and unambiguously identify different lineages when testing the lineage potency of cultured ESC carrying homozygous null mutations, particularly if relying only on gene expression analysis determined by Reverse Transcription-Polymerase Chain Reaction (RT-PCR). Given that many markers in use to establish definitive endoderm are also expressed in the VE (e.g., *Foxa2, Cer1, Hex, Sox17*), it is clear that being able to distinguish between these two tissue types is very important for developing models of differentiation for derivatives of these endodermal lineages. A full-expression profile for marker genes may not become available in the short term, but using a cohort of markers for a particular lineage coupled with morphological data may benefit future studies aimed at developing *in vitro* models of ESC-derived differentiated cell types.

**Table 1**

**Markers of Endoderm: Embryonic and Extraembryonic**

| | HB, 4–5 dpc | PE, ~4.5 dpc | VE, ~5–7 dpc | VxE, ~5.0 dpc | DE | Comment | Ref. |
|---|---|---|---|---|---|---|---|
| PE Marker | | | | | | | |
| Follistatin | ND | + | – | – | – | E, Me | 71 |
| PTHrP receptor* | ND | + | + | – | ND | | 31 |
| PDGF A | ND | + | ND | + | ND | E | 72 |
| PDGF α and β receptor | ND | + | ND | + | ND | E | 72 |
| tPA* | ND | + | – | – | ND | | 34,35 |
| Snail | – | + | – | – | + | Me | 73 |
| SPARC | ND | + | – | – | – | | 32,74 |
| Thrombomodulin | ND | + | – | – | – | TB, Me | 36 |
| VE Marker | | | | | | | |
| AFP | ND | – | + | + | – | | 22 |
| Amn | + | – | + | + | – | | 75 |
| BMP-2 | ND | – | + | ND | + | Me | 50 |
| COUP-TF I | + | ND | ND | ND | ND | | 76 |
| vHNF1 | + | + | + | + | + | | 49 |
| HNF4 | + | – | + | + | + | | 77 |
| Ihh | ND | – | + | + | – | | 51 |
| Maspin* | – | – | + | + | ND | | 78 |
| Msg1 | ND | ND | + | + | – | Me | 79 |
| uPA* | ND | – | + | – | ND | | 34,35 |
| Sfrp5 | ND | – | + | – | + | | 80 |
| Transferrin | ND | + | + | + | ND | TB | 81 |
| Ttr** | ND | – | + | + | + | | 82 |
| Villin | ND | – | + | + | – | | 32 |
| Endoderm Marker | | | | | | | |
| Cer1 | ND | – | + | – | + | | 29 |
| Foxa2 (HNF-3β) | ND | – | + | + | + | Me | 30 |
| Hex | ND | – | + | – | + | | 83 |
| Hesx1 | ND | – | + | – | + | E | 83 |
| **Sox17** | ND | + | – | + | + | | 70 |

This table is based on common markers used both *in vivo* and *in vitro* to detect distinct endodermal lineages as determined by *in situ* hybridization studies unless otherwise stated. Note: Precise timing of formation of HB, PE, VE, and VxE is poorly understood. dpc for the formation of these tissues was derived from these sources[13,14,20,21]. Symbols: +, Detected; –, Not detected; ND, not determined; *, based on protein expression studies; **, reported in rat.

In 'comment' column, expression in other tissues is listed in abbreviated form as follows: E, epiblast or ectoderm; Me, primitive streak or mesoderm; TB, trophoblast and/or trophectoderm.

Abbreviations: Lineages — definitive endoderm (DE), hypoblast (HB), parietal endoderm (PE), visceral endoderm (VE), visceral extraembryonic endoderm (VxE). Factors — α-fetoprotein (AFP), amnionless (Amn), chicken ovalbumin upstream promoter-transcription factor I (COUP-TF I) , Indian hedgehog (Ihh), hepatocyte nuclear factor 4 (HNF4), variant hepatocyte nuclear factor 1 (vHNF1), melanocyte-specific gene 1 (Msg 1), melanocyte-specific related gene 1 (MRG-1), parathyroid hormone-related peptide receptor (PTHrP receptor), platelet-derived growth factor (PDGF), secreted protein acidic and rich in cysteine (SPARC), tissue-type plasminogen activator 79 kDa (tPA), urokinase plasminogen activator 48 kDa (uPA), secreted frizzled-related protein 5 (Sfrp5), transthyretin (Ttr).

## CONCLUSION

This is the first attempt to characterize all the endodermal lineages (embryonic and extraembryonic) focusing specifically on the early conceptus. Although the ultrastructure of the HB was reported nearly 30 years ago[13,14], it has had little attention. This appears to be changing as studies aimed at identifying the factors involved in the first three lineage allocations in the mouse are now being reported. Currently there are no specific markers for the HB, which is hindering progress to understand its formation, derivatives, and role. A subtractive hybridization or microarray study comparing PB and HB would be most advantageous.

Over 30 years ago, the formation of the first PE cells with extensive filopodia were observed by Enders and colleagues[14] in close association with the lateral mural TE before a continuous layer of HB was generated. This would suggest that PE cells may arise from HB under the inductive influences of the TE, this remains an intriguing, but currently unexplored, notion.

As a result of its emerging key role in anteroposterior patterning, our knowledge of the VE has benefited enormously from gene-targeting studies that result in disruption of this lineage. Dissecting specification from differentiation of this lineage, however, would be aided greatly by VE-specific markers that are not expressed in the endoderm or other lineages. Complete developmental expression profiles for the markers in current use for the endodermal lineages have not been undertaken in most cases, and many markers display overlapping expression domains in the early conceptus. While *COUP-TF I* and *amn* are potential candidates as HB-specific markers, the expression of the former factor in other endodermal lineages of the early conceptus has not been reported and the latter factor is strongly expressed in the VE. All the current markers in use to identify VE including *Foxa2*, *vHNF1* and *HNF4* are expressed in other tissues including the endoderm. The putative endoderm–specific marker, *Sox17*, is also expressed at the same developmental stage in PE and VxE. It is clear that lineage-specific markers are required to resolve some fundamental questions about cell lineage.

## ACKNOWLEDGMENTS

The author wishes to thank Prof. Lynne Selwood, Dr. Lorraine Robb, and Dr. Don Newgreen for critical reading of the manuscript.
